# Chloride of Sodium in Intermittent Fever

**Published:** 1854-07

**Authors:** Edward D. G. Smith


### *Chloride of Sodium in Intermittent Fever.*

BY EDWARD D. G. SMITH, M. D.

Every one who has treated Intermittent Fever to any extent, has been annoyed by the obstinacy with which it sometimes resists all measures which are brought to bear upon it; and will be prepared to welcome what appears to be a new remedy. For though Chloride of Sodium has long been used in medicine for various purposes,—emetic, alterative, astringent, anthelmintic, &c., its use as an anti-periodic, as far as I can discover from considerable search and enquiry, is of modern date. Eberly, Dewees, Watson, Wood and Gregory,, in their several treatises upon Intermittent Fever do not mention it: neither is this property ascribed to it by Pereira or the U. S. Disepnsatory. The merit of first proposing it in this complaint, according to an article in the New Jersey Medical Reporter for December, 1851, is due to Dr. Piorry of Paris, who administers it in doses of two table spoonsful once or twice a day, and asserts that it arrests the paroxysms as promptly as quinine. Professor Herrick of Rush Medical College, also reports the result of several trials made with it, which go to corroborate the statements of Piorry. He prescribes it in doses of three or four drachms, twice daily in mucilage. After the fever is checked he gives it in smaller doses—ten grains with the same quantity of carbonate of iron two or three times daily, as a tonic and corrective of the secretions of the alimentary tube.—From the high terms in which it was spoken of by these gentlemen, I resolved to administer it upon the first opportunity. I did so and the result was so satisfactory, that I was induced to give it a more extensive trial. I have given it in many cases; in some of which quinine and arsenic had failed; and its almost invariable sucess, I think, warrants the praises bestowed upon it, and entitles it to recognition among the articles generally considered as specifics in this complaint. In fact, I do not recollect a single instance in which it has failed, when it has been faithfully taken according to my directions. In those cases in which it has appeared to fail, I have found upon strict enquiry, that the patient neglected to to take it,—generally because of its nauseous taste; which unfortunately cannot be entirely hidden, and which is the principal objection to its use, especially for children. I think there is less tendency to the return of the disease at the expiration of a week, when cured by the chloride, than when quinine or arsenic is used. My friends, Drs. Eyrich and L. G. Thomas, who have both given it successfully in many cases, are of the same opinion.

I found the doses directed by Drs. Piorry and Herrick rather too large;—in most instances the stomach being unable to retain them.—My method, therefore, has been to give it, during the interval of the fever, in doses of a *teaspoonful*—in weight from ʒjss to ʒij., in slippery elm or toast tea, every three hours for five or six times;—after thoroughly clearing out the alimentary canal by Calomel and Jalap or the Bilious Pills. Indeed, these doses have in many instances, caused vomiting at first; especially if there was much gastric derangement:—tolerance was generally soon established, and the vomiting ceased after the first or second dose, having undoubtedly done much good by clearing out the stomach. In a few cases, however. the medicine continued to cause vomiting at every dose, and I was obliged to discontinue it and resort to quinine or arsenic in its stead.

As some patients would doubtless object to this remedy in its natural state, it occurred to me that it would be well to disguise it; which I have been in the habit of doing by rubbing it up well with Red Saunders, thus making it of a fine pink color, and then adding a few drops of oil of anise. This deception has been successful. I have frequently been amused by comments upon, and questions as to the cause of its "saltish taste." The information that the taste was the result of the manner in which the medicine was prepared, has always been satisfactory,—no suspicions as to its real character, having, as far as I am aware, been entertained after this explanation.

The first patient to whom I administered the chloride was an old Irish woman of about 60, who had been afflicted with a tertian fever for some two months, and said she had taken quinine nearly every day since she had been sick:—a statement which I received with some allowance. She was in a very desponding frame of mind, and said she knew she was going to die, and all she wanted of the doctor was to give her a line to the priest, who refused to come to her spiritual aid without such a testimonial. I endeavored to encourage her, and induce her to make one more effort before abandoning herself to despair; assuring her that if I found she could not be cured, I would give her the desired epistle in time to secure her salvation before she died. After some persuasion she consented, and after the operation of a dose of bilious pills, which she took at bed-time, she commenced the next morning—it being her well day—with the chloride, in doses of a teaspoonful in toast tea, every three hours, with orders to soak her feet in hot water the next day, before the time when the paroxism generally came on, and to remain warmly covered up in bed during the day. She complied faithfully with my directions, though as she afterwards informed me, rather to secure the line to the priest, which I had refused to give her otherwise, than from any great faith in the medicine. To her and my great delight, she had not another paroxysm ; and I saw her again a month or two afterwards, when she told me she had not been sick a moment since she took the medicine, in praise of which she was eloquent. In fact, she had improved wonderfully in appearance and also in spirits: all of which improvement she ascribed to the chloride.

Her grand-daughter, a girl of 17 or 18, was afterwards attacked by the fever, also of the tertian type, and the old lady requested me to give her some of the same medicine. I accordingly left her a dose, to be preceded, as in the case of the grandmother, by three bilious pills over night. She took the pills (which operated thoroughly) and placed the powder under her pillow, where it remained when I visited her the next day, which was her well day. She and the old lady were both fully convinced that she was cured by having the medicine under her pillow, and I could not pursuade her that it would be necessary to take it that day. But unfortunately from the reputation of the chloride as a worker of miracles, this marvellous cure did not prove to be permanent,—the fever returning at its regular time the next day. It however, eventually yielded to the internal exhibition of the remedy, in which the confidence of all parties, especially myself—remained undiminished by the first failure. In fact, in every case in which I have administered it, my confidence in its virtues has been increased: the only instances in which it was at all shaken, which only occurred in my earlier experiments, being those cases of apparent failure already mentioned. I think, therefore, that it is not claiming too much for it to rank it with quinine and arsenic, the remedies most used in intermittent fever; and it possesses some advantages over either. It may be used more freely than either. It never causes the fulness of the head, and deafness which quinine sometimes does, especially when given in large doses:—or the gastric derangement, œdema, &c., which occasionally arise from the use of arsenic. It is certainly true that both of these are perfectly safe when properly used. The chloride has also the merit of *cheapness*, which is a recommendation, especially when the medicine, as so frequently happens, is to be given away.

As the chloride has so marked an influence over intermittent fever, it would be well to try its effect in the analogous disease neuralgia, and I intend doing so when a fitting opportunity presents. My experience with it thus far in this complaint, is of rather a negative character. I gave it to a woman for whom I had frequently prescribed, for tic-doloreux, and told her if it did not cure her to come to me again, which she promised to do, and I think would have done if she had not been cured ; as she was always troubling me before.— I never saw her again. However, I will leave others to draw the inference.—*N. Jersey Med. Reporter.*

